# The 3-Minute Psychomotor Vigilance Test Demonstrates Inadequate Convergent Validity Relative to the 10-Minute Psychomotor Vigilance Test Across Sleep Loss and Recovery

**DOI:** 10.3389/fnins.2022.815697

**Published:** 2022-02-15

**Authors:** Caroline A. Antler, Erika M. Yamazaki, Courtney E. Casale, Tess E. Brieva, Namni Goel

**Affiliations:** Biological Rhythms Research Laboratory, Department of Psychiatry and Behavioral Sciences, Rush University Medical Center, Chicago, IL, United States

**Keywords:** Psychomotor Vigilance Test, sleep deprivation, recovery, behavioral attention, repeated measures correlation, convergent validity, lapses, response speed

## Abstract

The Psychomotor Vigilance Test (PVT) is a widely used behavioral attention measure, with the 10-min (PVT-10) and 3-min (PVT-3) as two commonly used versions. The PVT-3 may be comparable to the PVT-10, though its convergent validity relative to the PVT-10 has not been explicitly assessed. For the first time, we utilized repeated measures correlation (rmcorr) to evaluate intra-individual associations between PVT-10 and PVT-3 versions across total sleep deprivation (TSD), chronic sleep restriction (SR) and multiple consecutive days of recovery. Eighty-three healthy adults (mean ± SD, 34.7 ± 8.9 years; 36 females) received two baseline nights (B1-B2), five SR nights (SR1-SR5), 36 h TSD, and four recovery nights (R1-R4) between sleep loss conditions. The PVT-10 and PVT-3 were completed every 2 h during wakefulness. Rmcorr compared responses on two frequently used, sensitive PVT metrics: reaction time (RT) *via* response speed (1/RT) and lapses (RT > 500 ms on the PVT-10 and > 355 ms on the PVT-3) by day (e.g., B2), by study phase (e.g., SR1-SR5), and by time point (1000–2000 h). PVT 1/RT correlations were generally stronger than those for lapses. The majority of correlations (48/50 [96%] for PVT lapses and 38/50 [76%] for PVT 1/RT) were values below 0.70, indicating validity issues. Overall, the PVT-3 demonstrated inadequate convergent validity with the “gold standard” PVT-10 across two different types of sleep loss and across extended recovery. Thus, the PVT-3 is not interchangeable with the PVT-10 for assessing behavioral attention performance during sleep loss based on the design of our study and the metrics we evaluated. Our results have substantial implications for design and measure selection in laboratory and applied settings, including those involving sleep deprivation.

## Introduction

One of the most commonly utilized measures in sleep research is the Psychomotor Vigilance Test (PVT), a measure of vigilant attention that requires participants to rapidly respond to visual cues randomly presented within specified interstimulus intervals (ISIs) without incorrectly responding when no stimulus is present (Dinges and Powell, 1985; Basner and Dinges, 2011). The PVT is often considered a “gold standard” measure of sleep loss deficits and it is one measure by which biomarkers or predictors of such deficits are compared (Dawson et al., 2014; Basner et al., 2015; Grandner et al., 2018; Moreno-Villanueva et al., 2018). The 10-min PVT (PVT-10) is the standard version, but more recently shorter 5-min (PVT-5) and 3-min (PVT-3) versions have been developed, particularly for applied settings that have limited time for testing (Loh et al., 2004; Basner et al., 2011).

Two published studies have directly compared performance on the PVT-10 and PVT-3 in response to sleep loss without using any other experimental manipulations: (1) the PVT-3 development study (Basner et al., 2011), which compared the PVT-10 (computer-based) and PVT-3 (handheld device-based) across total sleep deprivation (TSD) and five nights of 4 h time-in-bed (TIB) sleep restriction (SR) and (2) a validation study of smartphone-based and tablet-based 3-min PVT versions, which were compared to a laptop-based PVT-10 following 38 h TSD (Grant et al., 2017). Grant et al. (2017) reported significantly faster reaction times (RTs) and fewer lapses (PVT-10: >500 ms RT; PVT-3: >355 ms RT) on the PVT-3 relative to the PVT-10. Basner et al. (2011) also reported significantly faster RTs on the PVT-3, though they found fewer lapses on the PVT-3 only when 500 ms RT, and not 355 ms RT, was used as the lapse threshold for both PVT versions. In a study without sleep loss, Jones et al. (2018) compared performance on the PVT-10, PVT-3, and PVT-5 on the same device across 7 days in elite female basketball players and found that participants had significantly faster RTs and fewer lapses on the PVT-3 relative to the PVT-10 (and PVT-5). Additionally, a recent study involving sleep deprivation, alcohol consumption, and rest in a pressure chamber to simulate in-flight conditions compared performance on a personal computer-based PVT-10 and a handheld computer-based PVT-3 (Benderoth et al., 2021). Benderoth et al. (2021) determined that the two PVT versions had good parallel form reliability for 1/RT and lower, but still significant, correlations were found for number of lapses. Three of these studies concluded that the PVT-3 was a valid alternative to the PVT-10 (Basner et al., 2011; Grant et al., 2017; Benderoth et al., 2021), while the fourth concluded the tests were not interchangeable (Jones et al., 2018). Thus, further research is needed to systematically compare the PVT-10 and PVT-3 using the same device in highly controlled sleep loss studies.

Though averaging data from multiple time points may be necessary to meet various statistical assumptions, doing so can result in the loss of important data relating to changes in performance across time. Of note, the aforementioned sleep loss studies comparing the PVT-10 and PVT-3 (Basner et al., 2011; Grant et al., 2017) utilized averaged data in many of their analyses, with both studies using different numbers of averaged time points, and neither study examining time-of-day variation during baseline or recovery. As a result, any information relating to discrepancies between the measures at various time points, due to possible time-of-day variation or increased homeostatic sleep pressure (Gundel et al., 2007; Fimm et al., 2015), is missing from these studies. Thus, it is important to examine individual time points to determine time-of-day variation in performance, when comparing the PVT-10 and PVT-3.

Little is known about PVT performance across extended recovery periods (e.g., more than one consecutive recovery night) following sleep deprivation (Yamazaki et al., 2021b). Some (Lamond et al., 2008; Moreno-Villanueva et al., 2018; Yamazaki et al., 2021b, 2022b) but not all studies (Wehrens et al., 2012) have demonstrated that PVT-10 performance returns to baseline levels following one night of recovery sleep after TSD. PVT-10 performance recovery following SR is more complex, with studies reporting mixed findings; these include a failure to completely return to baseline, a delayed return to baseline requiring more than one recovery night, or a return to baseline after one recovery night (Dinges et al., 1997; Banks et al., 2010; Pejovic et al., 2013; Yamazaki et al., 2021b). PVT-3 performance also returns to baseline after one night of TSD (Yamazaki et al., 2021a), but data on PVT-3 performance across recovery periods after SR are lacking. Furthermore, no studies to date have directly compared the profile of PVT-10 and PVT-3 performance across an extended recovery period of long duration (e.g., multiple consecutive nights of 12 h) after sleep loss.

Given that prior studies found significant differences between the PVT-3 and PVT-10, that no sleep loss studies administered both versions on the same device or included an extended recovery period, that most analyses utilized averaged data, and that the PVT-3 is increasingly utilized (Basner and Rubinstein, 2011; Basner et al., 2011, 2018; Hilditch et al., 2016; Grant et al., 2017; Behrens et al., 2019; Hansen et al., 2019; Yamazaki et al., 2021a), there is a significant need for studies that compare the PVT-3 to the PVT-10 on the same device in the context of different types of commonly experienced sleep loss (TSD and SR) and with an extended recovery period. Further, no study to date has evaluated the convergent validity of the PVT-3 relative to the PVT-10 while considering repeated measurements (1) across an entire sleep deprivation study, (2) across an extended recovery period, or (3) with the measures administered on the same device.

The current study utilized the repeated measures correlation (rmcorr) technique (Bland and Altman, 1995) to examine for the first time the intra-individual (within-subject) association between the PVT-10 and PVT-3 across time. This statistical method reveals the common intra-individual linear relationship, which is considered representative of the convergent validity of the measures between PVT-10 and PVT-3 metrics. Assuming the PVT-3 and PVT-10 measures are comparable in their ability to assess performance and detect change across time, it was hypothesized that relatively strong rmcorr effect sizes for comparisons between the measures for PVT lapses and PVT 1/RT would be detected, and that these relationships would remain strong regardless of time of day, since the measures should comparably capture any variations in performance due to time effects. It was also hypothesized that all correlations would be stronger for PVT 1/RT relative to PVT lapses as well as stronger during sleep deprivation relative to baseline or recovery periods for both PVT lapses and PVT 1/RT. Lastly, it was hypothesized that correlation patterns for both PVT lapses and PVT 1/RT across the extended recovery period would not differ between those exposed to TSD versus those exposed to SR prior to recovery.

## Materials and Methods

### Participants

Eighty-three healthy adults were recruited in response to study advertisements. Participants reported habitual nightly sleep durations between 6.5 and 8.5 h, with habitual bedtimes between 2200 and 0000 h, and habitual awakenings between 0600 and 0930 h; these were confirmed *via* wrist actigraphy prior to study entry. Participants did not engage in habitual napping and did not present with a sleep disorder. They did not have any acute or chronic psychological and medical conditions. Participants did not take regular medications (except for oral contraceptive use in females) and were non-smokers with body mass index values between 17.3 and 30.9 kg/m^2^. See Yamazaki et al. (2021b) for additional details on recruitment methods, inclusion and exclusion criteria, sample characteristics, general study procedures, and participant monitoring. The protocol was approved by the University of Pennsylvania’s Institutional Review Board. All participants received compensation for their participation and provided written informed consent in accordance with the Declaration of Helsinki.

### Procedures

Participants engaged in a 13-day laboratory study during which they received daily checks of vital signs and symptoms by nurses (with a physician on call). The 13-day study consisted of two baseline nights (B1-B2, 10 h [2200–0800 h] and 12 h [2200–1000 h] TIB, respectively) followed by randomization to either five nights of 4 h TIB SR (SR1-SR5, 0400–0800 h, *N* = 41; Condition A) or 36 h TSD (wakefulness from 1000 to 2200 h the following day, *N* = 42; Condition B), both of which were followed by four nights of 12 h TIB (2200–1000 h) recovery sleep (R1-R4). After R1-R4, participants in the initial SR condition (Condition A) were exposed to 36 h TSD and those in the initial TSD condition (Condition B) were exposed to five nights of 4 h TIB SR. Participants were randomized in groups of four and blinded to their condition assignment until the evening after the second baseline night.

A computer-based neurobehavioral test battery was administered every 2 h during wakefulness throughout the study. Between test bouts participants were ambulatory and permitted to perform sedentary activities; however, they were not allowed to exercise. Ambient temperature was maintained between 22 and 24°C. Laboratory light levels remained constant at <50 lux during scheduled wakefulness and <1 lux during scheduled sleep periods (Yamazaki and Goel, 2020; Brieva et al., 2021; Yamazaki et al., 2021b, 2022a; Casale et al., 2022).

### Neurobehavioral Measures

The computer-based neurobehavioral test battery included two widely used versions of a measure of behavioral attention: the 10-min PVT (Lim and Dinges, 2008; Basner and Dinges, 2011) and the 3-min PVT (Basner et al., 2011). Both PVT tests were administered in an environment with minimal distractions. The PVT-10 was administered before the PVT-3 during all test bouts for all participants. Participants were instructed to hit the space bar as quickly as possible after they were presented with a visual cue on the screen. Visual cues were randomly presented within specified interstimulus intervals (ISIs, or the period between the previous response and the next stimulus) specific to each measure version; the PVT-10 ISI was 2–10 s while the PVT-3 ISI was 1–4 s (Basner et al., 2011). Outcome measures were the number of lapses [RT > 500 ms on the PVT-10 and > 355 ms on the PVT-3 (Basner et al., 2011)] and response speed (mean 1/RT, henceforth referred to as 1/RT).

### Statistical Analysis

Although repeated measures data are inherently valuable, their analyses can be challenging due to frequent violation of the assumptions of various statistical procedures (Keselman et al., 2001; Park et al., 2009; Bakdash and Marusich, 2017). The methods for correcting these violations, such as averaging, can result in the loss of otherwise meaningful data (Bland and Altman, 1995), and conducting analyses despite violations can result in misleading or uninterpretable results (Glass et al., 1972; Hubbard, 1978; Kenny and Judd, 1986; Scariano and Davenport, 1987). As such, instead of using Pearson’s correlations, we used repeated measures correlations [rmcorr (Bakdash and Marusich, 2017; Bakdash and Marusich, 2020)], to compare PVT-10 lapses to PVT-3 lapses and to compare PVT-10 1/RT to PVT-3 1/RT. Of note, we specifically used correlational analyses because convergent validity is exclusively assessed *via* correlation (Chin and Yao, 2014). Rmcorr analyses were conducted by day (e.g., B2, SR1, R3, etc.), by study phase (e.g., SR1-SR5, R1-R4, etc.), and by time point (e.g., 1000 h, 1200 h, etc.) across the entire 13-day study and across recovery only (R1-R4) for Condition A and Condition B using the rmcorr R package (Bakdash and Marusich, 2020). By day analyses included data from the 1000–2000 h time points for B2 and for R1-R4. To retain as much data as possible, by day analyses for SR1-SR4 included early morning and late-night time points (e.g., 0800–0200 h the day after each night of SR). For SR5, only the 0800 h through 2000 h time points were collected given the start of R1 occurred immediately after SR5. TSD day was defined as 2200 h on the night of TSD through 2000 h the next day. By study phase analyses included all time points across each period (e.g., R1-R4). For Condition A and Condition B, the B2-R4 study phase included all time points from B2 through R4. The all-study days study phase included all time points from B2 through the end of TSD (2000 h) for Condition A and through the end of SR5 (2000 h) for Condition B.

Rmcorr confidence intervals (CIs) were determined using bootstrapping with replacement and using 1,000 samples (Shan et al., 2021). To meet rmcorr’s linearity assumption, PVT lapses were natural log transformed [nlog(lapses + 0.5)] for the by time point analyses to account for non-linear associations apparent with visual plot inspection (Cohen et al., 2003; Bakdash and Marusich, 2017). The False Discovery Rate correction of Benjamini-Hochberg (Benjamini and Hochberg, 1995) was applied to all rmcorr *p*-values to account for multiplicity (Gbyl et al., 2021), but notably, this did not alter the significance of any test. Thus, unadjusted *p*-values are reported. Rmcorr coefficient (*r*_*rm*_) magnitude was conservatively interpreted using the following ranges: 0.00–0.29, negligible; 0.30–0.49, weak; 0.50–0.69, moderate; 0.70–0.89, strong; and 0.90–1.00, very strong (Carlson and Herdman, 2010; Mukaka, 2012; Post, 2016; Fernández-Marcos et al., 2018; Schober et al., 2018; Yadav, 2018). Furthermore, as per recommendations for interpreting convergent validity coefficients (Carlson and Herdman, 2010; Post, 2016), *r*_*rm*_ values < 0.50 indicated the PVT-3 showed inadequate convergent validity with the PVT-10, *r*_*rm*_ values > 0.70 indicated adequate convergent validity between the measures and *r*_*rm*_ values between 0.50 and 0.70 indicated validity issues between the measures. All statistical analyses were conducted in the R software environment (R Core Team, 2020). All analyses were two-sided with a *p*-value < 0.05 considered statistically significant. No participants were excluded from the analyses. Pairwise deletion was used for all analyses to minimize data loss since single data points were missing at random throughout the study; the degrees of freedom (*df*) in Tables 1–4 serve as a proxy for the amount of data lost based on the formula *df* = *N*(*k*-1) – 1, where *N* is the total number of participants and *k* is the number of repeated measures per participant.

**TABLE 1:** PVT-10 and PVT-3 lapses rmcorr results by day and by study phase.

Condition	Statistic	Study day											Study phase				
		B2	SR1	SR2	SR3	SR4	SR5	R1	R2	R3	R4	TSD	ALL	B2-R4	SR1-SR5	R1-R4	TSD
A	*r*_*rm*_	0.134	0.395	0.430	0.388	0.420	0.398	0.523	0.178	0.145	0.293	0.489	0.566	0.535	0.472	0.334	0.489
	*df*	156	327	368	366	368	182	140	202	162	163	409	3345	2894	1779	793	409
	*p*	0.093	<0.001	<0.001	<0.001	<0.001	<0.001	<0.001	0.011	0.065	<0.001	<0.001	<0.001	<0.001	<0.001	<0.001	<0.001
	CI low	−0.005	0.299	0.315	0.284	0.304	0.227	0.105	−0.039	−0.057	0.112	0.422	0.534	0.499	0.423	0.228	0.422
	CI high	0.259	0.483	0.547	0.489	0.528	0.516	0.699	0.350	0.333	0.443	0.563	0.597	0.570	0.520	0.426	0.563
PVT-10	Mdn (IQR)	0.00 (1.00)	1.00 (3.00)	2.00 (5.00)	2.00 (6.00)	3.00 (7.00)	3.00 (8.00)	1.00 (3.00)	1.00 (3.00)	1.00 (3.00)	1.00 (3.00)	4.00 (11.00)	2.00 (5.00)	1.00 (4.00)	2.00 (6.00)	1.00 (3.00)	4.00 (11.00)
PVT-3	Mdn (IQR)	1.00 (3.00)	2.00 (7.00)	4.00 (9.00)	4.00 (9.00)	6.00 (10.00)	6.00 (14.25)	2.00 (5.00)	2.00 (6.00)	2.00 (6.00)	2.00 (6.00)	8.00 (14.00)	3.00 (9.00)	3.00 (9.00)	4.00 (10.00)	2.00 (6.00)	8.00 (14.00)

		**B2**	**TSD**	**R1**	**R2**	**R3**	**R4**	**SR1**	**SR2**	**SR3**	**SR4**	**SR5**	**ALL**	**B2-R4**	**TSD**	**R1-R4**	**SR1-SR5**

B	*r*_*rm*_	0.000	0.547	0.066	0.258	0.238	0.813	0.563	0.544	0.519	0.446	0.330	0.662	0.705	0.547	0.513	0.524
	*df*	149	388	166	209	167	167	335	377	377	377	186	3442	1449	388	838	1824
	*p*	1.000	<0.001	0.392	<0.001	0.002	<0.001	<0.001	<0.001	<0.001	<0.001	<0.001	<0.001	<0.001	<0.001	<0.001	<0.001
	CI low	−0.253	0.449	−0.182	0.002	−0.010	−0.279	0.439	0.414	0.412	0.338	0.147	0.633	0.661	0.449	0.067	0.469
	CI high	0.204	0.637	0.270	0.473	0.469	0.909	0.676	0.648	0.630	0.547	0.521	0.692	0.749	0.637	0.730	0.577
PVT-10	Mdn (IQR)	0.00 (1.00)	2.00 (7.00)	0.00 (1.00)	0.00 (1.00)	0.00 (1.00)	0.00 (1.00)	1.00 (3.00)	1.00 (4.25)	2.00 (6.00)	2.00 (9.00)	2.00 (6.00)	1.00 (4.00)	0.00 (2.00)	2.00 (7.00)	0.00 (1.00)	2.00 (5.50)
PVT-3	Mdn (IQR)	1.00 (3.00)	5.00 (12.00)	1.00 (3.00)	1.00 (3.00)	1.00 (3.00)	1.00 (3.00)	3.00 (8.00)	4.00 (8.00)	4.00 (10.00)	6.00 (12.00)	5.00 (11.00)	3.00 (8.00)	2.00 (5.00)	5.00 (12.00)	1.00 (3.00)	1.00 (10.00)

*PVT-10 = 10-min Psychomotor Vigilance Test; PVT-3 = 3-min Psychomotor Vigilance Test; rmcorr = repeated measures correlation; B2 = baseline day 2; SR1-SR5 = sleep restriction days 1 through 5; R1-R4 = recovery days 1 through 4; TSD = total sleep deprivation; ALL = all study days; CI = bootstrapped 95% confidence interval; Mdn = median; IQR = interquartile range.*

## Results

### Participant Characteristics

Eighty-three healthy adults (mean ± SD, 34.7 ± 8.9 years; 36 females) (aged 21–50 years, 72.3% African American; 43.4% female) participated in the study, with *N* = 41 participants randomly assigned to Condition A (SR first) and *N* = 42 participants randomly assigned to Condition B (TSD first). There were no significant differences between conditions in age, BMI, chronotype, or the percentage of participants who were female or African American (Yamazaki et al., 2021b). There were also no significant differences between conditions in pre-study actigraphic sleep duration, onset, offset, or midpoint, or in baseline polysomnographic total sleep time or sleep onset latency (Yamazaki et al., 2021b).

### Psychomotor Vigilance Test Lapses

**FIGURE 1:**
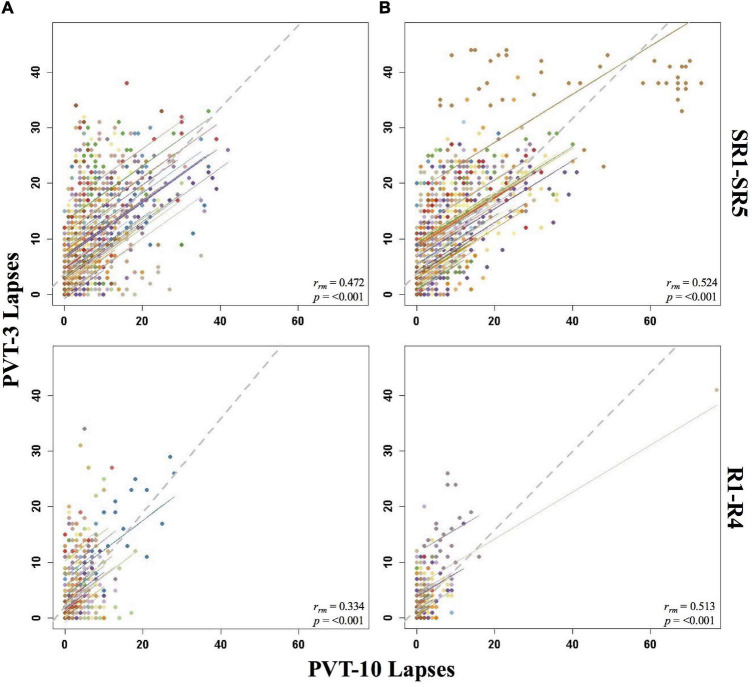
Rmcorr plots of repeated-measures correlations between 10-min Psychomotor Vigilance Test (PVT-10) and 3-min Psychomotor Vigilance Test (PVT-3) lapses by study phase for Condition A **(A)** and Condition B **(B)**. Each color represents a distinct participant with each point showing performance on both measures at one time point while the corresponding line shows the rmcorr fit for that participant (Bakdash and Marusich, 2020; R Core Team, 2020). The gray dashed line represents the regression line obtained by ignoring repeated measurements and treating the data as independent observations; *r*_*rm*_ represents the common within-individual association (rmcorr). Rmcorr effect sizes were interpreted as follows: 0.00–0.29, negligible; 0.30–0.49, weak; 0.50–0.69, moderate; 0.70–0.89, strong; and 0.90–1.00, very strong. Included time points for study phases were as follows: sleep restriction day one from 0800 h through sleep restriction day five at 2000 h (SR1-SR5) and recovery day one from 1000 h through recovery day four at 2000 h (R1-R4).

**FIGURE 2:**
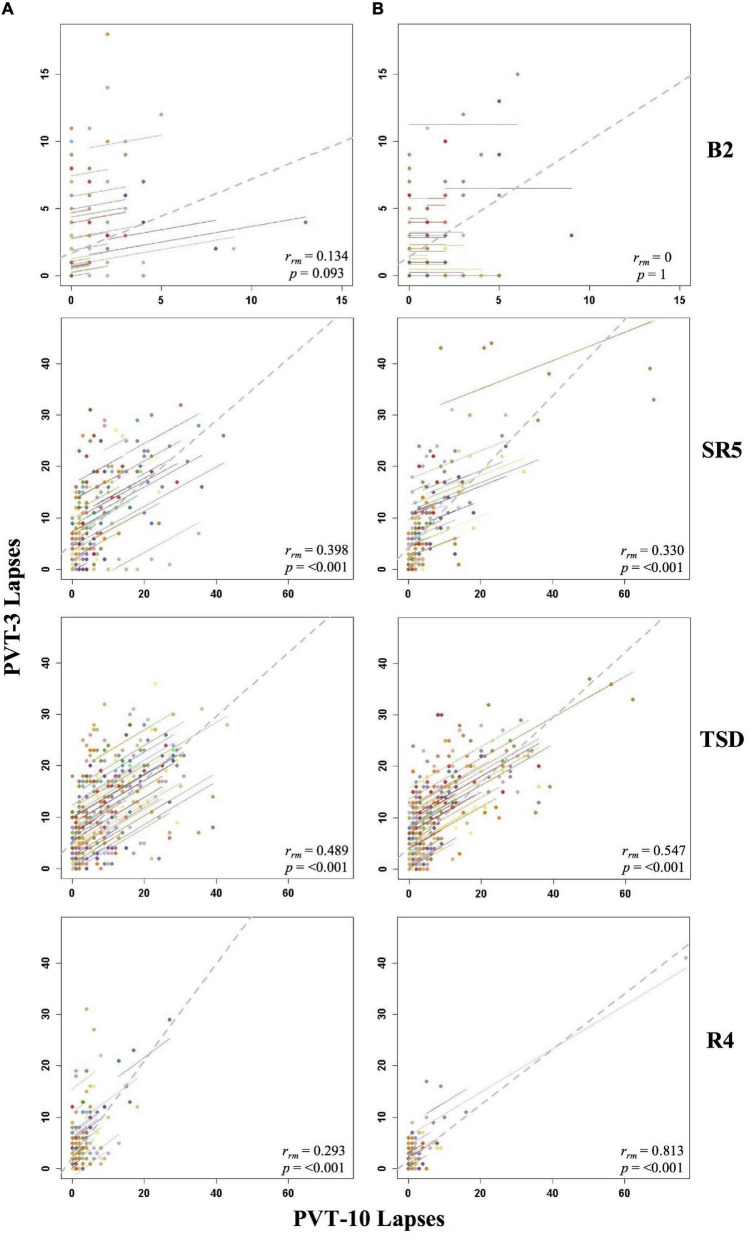
Rmcorr plots of repeated-measures correlations between 10-min Psychomotor Vigilance Test (PVT-10) and 3-min Psychomotor Vigilance Test (PVT-3) lapses by study day for Condition A **(A)** and Condition B **(B)**. Each color represents a distinct participant with each point showing performance on both measures at one time point while the corresponding line shows the rmcorr fit for that participant (Bakdash and Marusich, 2020; R Core Team, 2020). The gray dashed line represents the regression line obtained by ignoring repeated measurements and treating the data as independent observations; *r*_*rm*_ represents the common within-individual association (rmcorr). Rmcorr effect sizes were interpreted as follows: 0.00–0.29, negligible; 0.30–0.49, weak; 0.50–0.69, moderate; 0.70–0.89, strong; and 0.90–1.00, very strong. Included time points for each day were as follows: baseline day 2 (B2) from 1000 to 2200 h; sleep restriction day 5 (SR5) from 0800 to 2000 h; total sleep deprivation (TSD) from 2200 to 2000 h; and recovery day 4 (R4) from 1000 to 2000 h.

**FIGURE 3:**
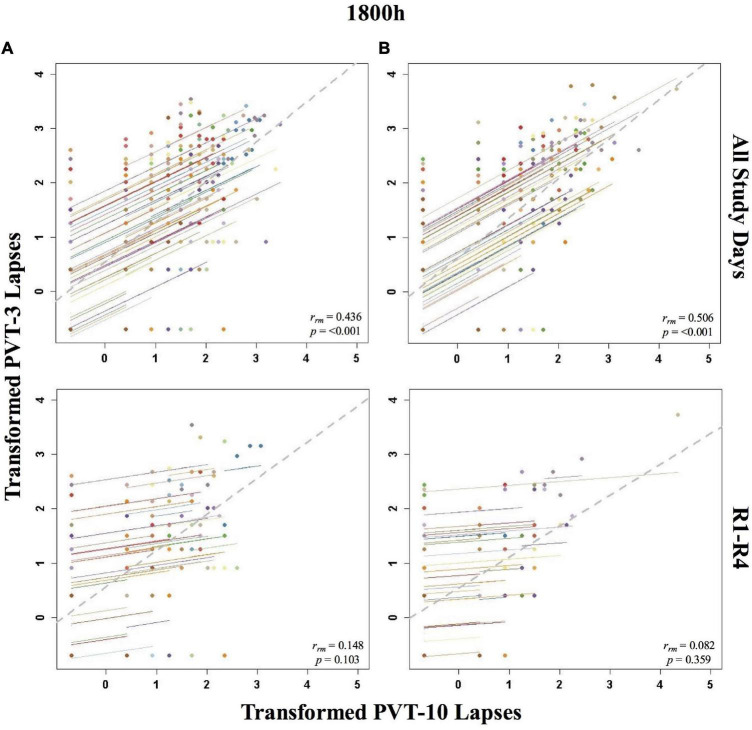
Rmcorr plots of repeated-measures correlations between 10-min Psychomotor Vigilance Test (PVT-10) and 3-min Psychomotor Vigilance Test (PVT-3) transformed lapses at 1800 h across the entire study (All Study Days) and across only recovery days 1–4 (R1-R4) for Condition A **(A)** and Condition B **(B)**. Each color represents a distinct participant with each point showing performance on both measures at one time point while the corresponding line shows the rmcorr fit for that participant (Bakdash and Marusich, 2020; R Core Team, 2020). The gray dashed line represents the regression line obtained by ignoring repeated measurements and treating the data as independent observations; *r*_*rm*_ represents the common within-individual association (rmcorr). Rmcorr effect sizes were interpreted as follows: 0.00–0.29, negligible; 0.30–0.49, weak; 0.50–0.69, moderate; 0.70–0.89, strong; and 0.90–1.00, very strong. Values were transformed by adding 0.5 and natural log transforming the result.

Tables 1, 2 show *r*_*rm*_, degrees of freedom, *p*-values, bootstrapped 95% CIs, and median and interquartile range (IQR) values for the PVT-3 and PVT-10 separately for the PVT lapses analyses. Median values were calculated for each value represented in the tables (i.e., 1000 h, B2, SR1-SR5, etc.) for all participants within each condition. We present medians, rather than means, since they are less susceptible to skewing by outliers and better reflect the central tendency of these data. Visualization is important for interpreting rmcorr results (Bakdash and Marusich, 2017; Schober et al., 2018), and as such, we have included select plots (Figures 1–3) as examples of the range of observed effects for each analysis type.

**TABLE 2:** PVT-10 and PVT-3 transformed lapses rmcorr results by time point.

Condition	Statistic	All study days					Recovery only				
Time		1000 h	1200 h	1600 h	1800 h	2000 h	1000 h	1200 h	1600 h	1800 h	2000 h
A	*r*_*rm*_	0.594	0.593	0.523	0.436	0.461	0.238	0.319	0.224	0.148	0.319
	*df*	387	409	405	408	383	100	122	119	121	122
	*p*	<0.001	<0.001	<0.001	<0.001	<0.001	0.016	<0.001	0.014	0.103	<0.001
	CI low	0.529	0.525	0.444	0.360	0.369	0.057	0.173	0.047	−0.020	0.126
	CI high	0.652	0.656	0.592	0.512	0.549	0.415	0.464	0.427	0.308	0.488
PVT-10*	Mdn (IQR)	1.00 (4.00)	1.00 (4.00)	1.00 (4.00)	1.00 (4.00)	1.00 (4.00)	1.00 (3.00)	1.00 (3.00)	1.00 (3.00)	1.00 (3.50)	1.00 (4.00)
PVT-3*	Mdn (IQR)	3.00 (9.00)	3.00 (8.5)	3.00 (8.00)	3.00 (8.00)	3.00 (8.00)	2.00 (5.75)	2.00 (5.00)	2.00 (5.00)	3.00 (5.00)	2.00 (6.00)
B	*r*_*rm*_	0.589	0.514	0.502	0.506	0.527	0.199	0.031	0.125	0.082	0.242
	*df*	419	418	419	419	389	125	124	125	125	125
	*p*	<0.001	<0.001	<0.001	<0.001	<0.001	0.025	0.733	0.163	0.359	0.006
	CI low	0.523	0.438	0.433	0.430	0.445	0.042	−0.175	−0.036	−0.136	0.094
	CI high	0.645	0.577	0.569	0.574	0.595	0.346	0.225	0.299	0.263	0.391
PVT-10*	Mdn (IQR)	1.00 (3.00)	1.00 (2.00)	1.00 (3.00)	1.00 (3.00)	1.00 (2.00)	0.00 (1.00)	0.00 (1.00)	0.00 (1.00)	0.00 (1.00)	0.00 (1.00)
PVT-3*	Mdn (IQR)	2.00 (7.00)	2.00 (6.00)	2.00 (6.00)	2.00 (5.75)	2.00 (6.00)	1.00 (3.00)	1.00 (3.00)	1.00 (3.00)	1.00 (3.00)	1.00 (3.00)

PVT-10 = 10-min Psychomotor Vigilance Test; PVT-3 = 3-min Psychomotor Vigilance Test; rmcorr = repeated measures correlation; CI = bootstrapped 95% confidence interval; Mdn = median; IQR = interquartile range. * = PVT lapses median and IQR values are not transformed.

#### By Study Phase

Overall, Condition B yielded stronger correlations relative to Condition A, and all the by study phase analyses for PVT lapses were significant (Table 1). The *r*_*rm*_ for B2-R4 was strong for Condition B and moderate for Condition A. SR1-SR5 and R1-R4 were weak for Condition A and moderate for Condition B. Interestingly, the entire study (all-study) *r*_*rm*_ was in the moderate range for both conditions. Figure 1 presents rmcorr plots for the SR1-SR5 and R1-R4 analyses for Condition A and Condition B.

#### By Day

The by day rmcorr analyses revealed a wide range of rmcorr coefficient values for PVT lapses across study days (Table 1). The only correlation that was strong was R4 for Condition B. For Condition A, only R1 demonstrated a moderate correlation. For Condition B, TSD and SR1-SR3 demonstrated moderate correlations. For Condition A, weak correlations were observed for SR1-SR5 and for TSD. For Condition B, only SR4 and SR5 demonstrated weak correlations. R2 correlations were in the negligible range for both conditions while R4 was negligible for Condition A and R3 was negligible for Condition B. Neither condition demonstrated significant correlations at B2 while R3 was non-significant for Condition A and R1 was non-significant for Condition B. Figure 2 presents B2, SR5, TSD, and R4 rmcorr plots for Condition A and Condition B. Notably, most individual lines approximate the overall regression line except for B2 for both conditions.

#### By Time Point

The entire study (all-study) duration time point rmcorr analyses for PVT lapses were all significant for Condition A and Condition B (Table 2). All *r*_*rm*_ values were moderate for Condition B, while only the 1000, 1200, and 1600 h time point correlations were moderate for Condition A (the 1800 and 2000 h time points were weak). The recovery (R1-R4) time point *r*_*rm*_ coefficients were weaker than the all-study time point coefficients. Across recovery for Condition A, the 1200 and 2000 h time point correlations were in the weak range while the 1000, 1600, and 1800 h time point correlations were in the negligible range or were non-significant. For Condition B, the all-study by time point correlations were negligible across recovery while the 1200, 1600, and 1800 h time point correlations were all non-significant. Figure 3 presents rmcorr plots for 1800 h by time point analyses as an example of moderate, weak and negligible *r*_*rm*_ correlations by time point across the entire study and across recovery for both conditions.

### Psychomotor Vigilance Test 1/RT

**FIGURE 4:**
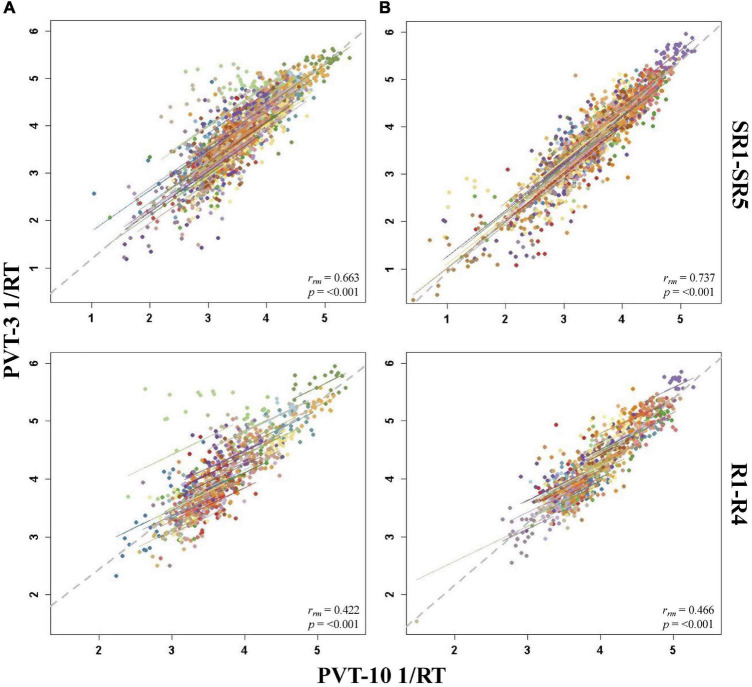
Rmcorr plots of repeated-measures correlations between 10-min Psychomotor Vigilance Test (PVT-10) and 3-min Psychomotor Vigilance Test (PVT-3) response speed (1/RT) by study phase for Condition A **(A)** and Condition B **(B)**. Each color represents a distinct participant with each point showing performance on both measures at one time point while the corresponding line shows the rmcorr fit for that participant (Bakdash and Marusich, 2020; R Core Team, 2020). The gray dashed line represents the regression line obtained by ignoring repeated measurements and treating the data as independent observations; *r*_*rm*_ represents the common within-individual association (rmcorr). Rmcorr effect sizes were interpreted as follows: 0.00–0.29, negligible; 0.30–0.49, weak; 0.50–0.69, moderate; 0.70–0.89, strong; and 0.90–1.00, very strong. Included time points were as follows: sleep restriction day one from 0800 h through sleep restriction day five at 2000 h (SR1-SR5) and recovery day one from 1000 h through recovery day four at 2000 h (R1–R4).

**FIGURE 5:**
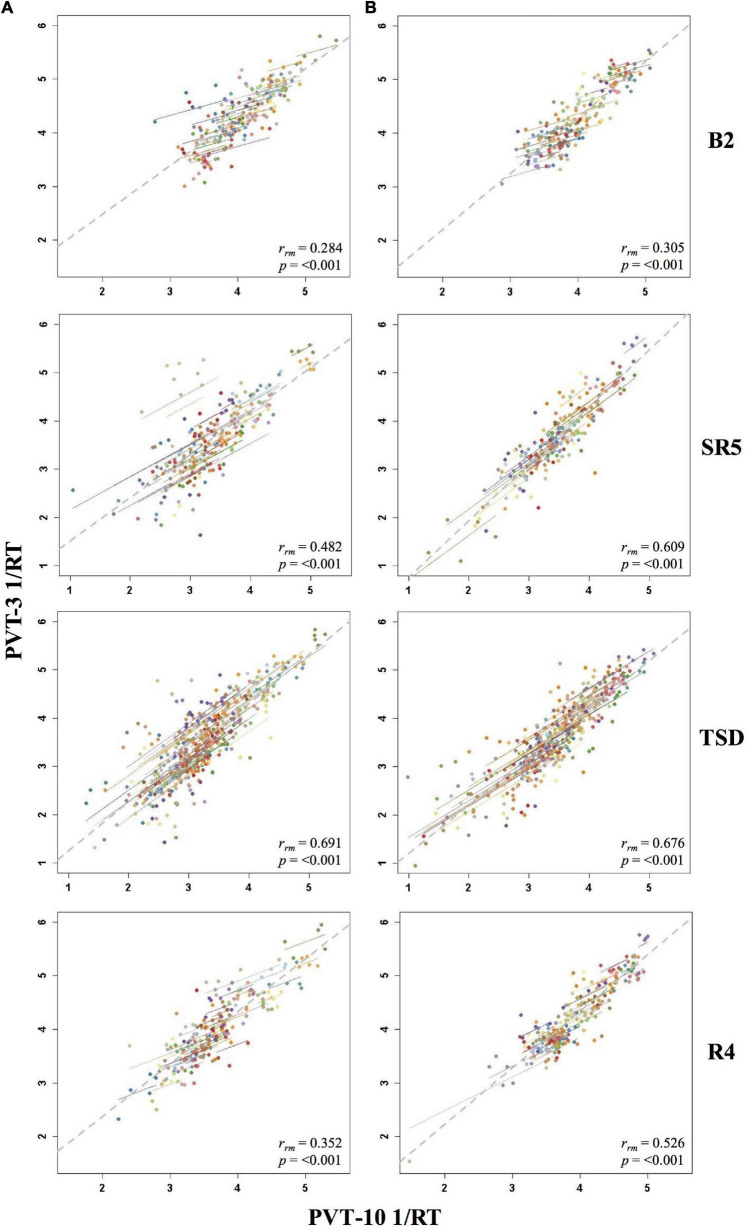
Rmcorr plots of repeated-measures correlations between 10-min Psychomotor Vigilance Test (PVT-10) and 3-min Psychomotor Vigilance Test (PVT-3) response speed (1/RT) by study day for Condition A **(A)** and Condition B **(B)**. Each color represents a distinct participant with each point showing performance on both measures at one time point while the corresponding line shows the rmcorr fit for that participant (Bakdash and Marusich, 2020; R Core Team, 2020). The gray dashed line represents the regression line obtained by ignoring repeated measurements and treating the data as independent observations; *r*_*rm*_ represents the common within-individual association (rmcorr). Rmcorr effect sizes were interpreted as follows: 0.00–0.29, negligible; 0.30–0.49, weak; 0.50–0.69, moderate; 0.70–0.89, strong; and 0.90–1.00, very strong. Included time points for each day were as follows: baseline day 2 (B2) from 1000 to 2200 h; sleep restriction day 5 (SR5) from 0800 to 2000 h; total sleep deprivation (TSD) from 2200 to 2000 h; and recovery day 4 (R4) from 1000 to 2000 h.

**FIGURE 6:**
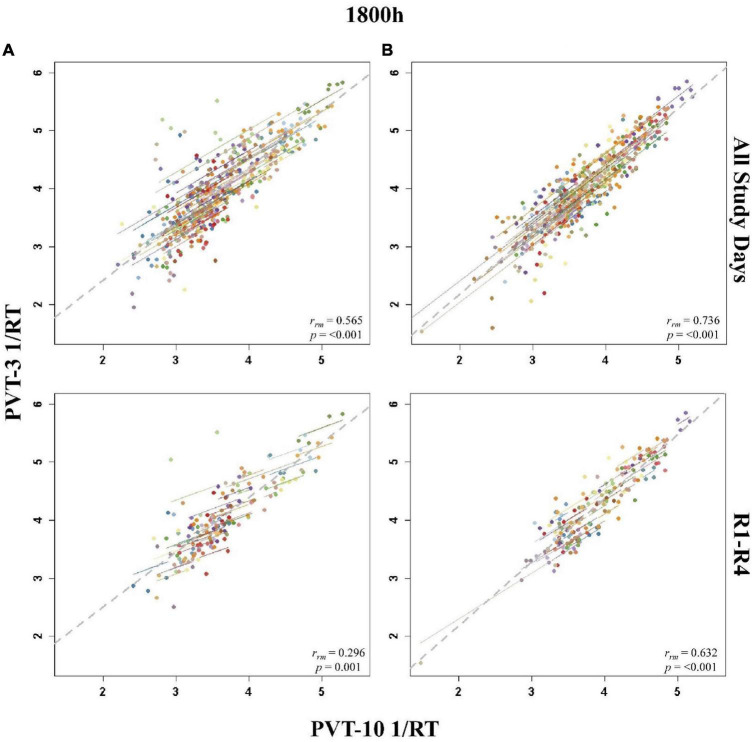
Rmcorr plots of repeated-measures correlations between 10-min Psychomotor Vigilance Test (PVT-10) and 3-min Psychomotor Vigilance Test (PVT-3) response speed (1/RT) at 1800 h across the entire study (All Study Days) and across only recovery days 1–4 (R1-R4) for Condition A **(A)** and Condition B **(B)**. Each color represents a distinct participant with each point showing performance on both measures at one time point while the corresponding line shows the rmcorr fit for that participant (Bakdash and Marusich, 2020; R Core Team, 2020). The gray dashed line represents the regression line obtained by ignoring repeated measurements and treating the data as independent observations; *r*_*rm*_ represents the common within-individual association (rmcorr). Rmcorr effect sizes were interpreted as follows: 0.00–0.29, negligible; 0.30–0.49, weak; 0.50–0.69, moderate; 0.70–0.89, strong; and 0.90–1.00, very strong.

Tables 3, 4 show *r*_*rm*_, degrees of freedom, *p*-values, bootstrapped 95% CIs, and median and IQR values for the PVT-3 and PVT-10 separately for the PVT 1/RT analyses. Median values were calculated for each value represented in the tables (i.e., 1000 h, B2, SR1-SR5, etc.) for all participants within each condition. As noted in section “Psychomotor Vigilance Test Lapses,” we present medians, rather than means, since they are less susceptible to skewing by outliers and better reflect the central tendency of these data. Select rmcorr plots as examples of the range of observed effects for each analysis type are included (Figures 4–6).

**TABLE 3:** PVT-10 and PVT-3 1/RT rmcorr results by day and by study phase.

Condition	Statistic	Study day											Study phase				
		B2	SR1	SR2	SR3	SR4	SR5	R1	R2	R3	R4	TSD	ALL	B2-R4	SR1-SR5	R1-R4	TSD
A	*r*_*rm*_	0.284	0.699	0.629	0.623	0.590	0.482	0.540	0.310	0.375	0.352	0.691	0.712	0.678	0.663	0.422	0.691
	*df*	156	327	368	366	368	182	140	202	162	163	409	3345	2894	1779	793	409
	*p*	<0.001	<0.001	<0.001	<0.001	<0.001	<0.001	<0.001	<0.001	<0.001	<0.001	<0.001	<0.001	<0.001	<0.001	<0.001	<0.001
	CI low	0.129	0.627	0.543	0.539	0.523	0.328	0.391	0.146	0.232	0.189	0.633	0.690	0.650	0.632	0.357	0.633
	CI high	0.451	0.762	0.702	0.695	0.655	0.598	0.663	0.463	0.499	0.484	0.741	0.733	0.702	0.696	0.481	0.741
PVT-10	Mdn (IQR)	4.03 (0.75)	3.70 (0.84)	3.60 (0.80)	3.50 (0.78)	3.36 (0.81)	3.29 (0.87)	3.65 (0.85)	3.63 (0.73)	3.64 (0.69)	3.62 (0.60)	3.21 (0.80)	3.54 (0.84)	3.58 (0.83)	3.49 (0.84)	3.63 (0.72)	3.21 (0.80)
PVT-3	Mdn (IQR)	4.31 (0.78)	4.01 (1.05)	3.82 (1.14)	3.76 (1.02)	3.56 (1.04)	3.59 (1.09)	4.13 (0.97)	3.95 (1.01)	4.03 (0.93)	3.92 (0.93)	3.49 (1.13)	3.85 (1.08)	3.91 (1.05)	3.76 (1.10)	4.02 (0.98)	3.49 (1.13)

		**B2**	**TSD**	**R1**	**R2**	**R3**	**R4**	**SR1**	**SR2**	**SR3**	**SR4**	**SR5**	**ALL**	**B2-R4**	**TSD**	**R1-R4**	**SR1-SR5**

B	*r*_*rm*_	0.305	0.676	0.408	0.376	0.370	0.526	0.747	0.744	0.691	0.691	0.609	0.808	0.784	0.676	0.466	0.737
	*df*	149	388	166	209	167	167	335	377	377	377	186	3442	1449	388	838	1824
	*p*	<0.001	<0.001	<0.001	<0.001	<0.001	<0.001	<0.001	<0.001	<0.001	<0.001	<0.001	<0.001	<0.001	<0.001	<0.001	<0.001
	CI low	0.152	0.611	0.272	0.217	0.228	0.164	0.688	0.670	0.620	0.616	0.485	0.791	0.757	0.611	0.353	0.706
	CI high	0.465	0.734	0.521	0.498	0.508	0.695	0.798	0.807	0.754	0.757	0.709	0.823	0.810	0.734	0.569	0.767
PVT-10	Mdn (IQR)	3.88 (0.74)	3.48 (0.84)	3.89 (0.74)	3.88 (0.78)	3.90 (0.82)	3.83 (0.72)	3.60 (0.80)	3.55 (0.76)	3.50 (0.78)	3.42 (0.84)	3.45 (0.79)	3.64 (0.80)	3.78 (0.78)	3.48 (0.84)	3.88 (0.76)	3.50 (0.81)
PVT-3	Mdn (IQR)	4.12 (0.97)	3.61 (1.07)	4.21 (0.98)	4.16 (0.95)	4.23 (0.98)	4.13 (0.93)	3.82 (1.03)	3.75 (0.91)	3.70 (0.99)	3.59 (1.02)	3.60 (0.95)	3.87 (1.00)	4.04 (0.95)	3.61 (1.07)	4.17 (0.95)	3.71 (0.99)

*PVT-10 = 10-min Psychomotor Vigilance Test; PVT-3 = 3-min Psychomotor Vigilance Test; 1/RT = response speed; rmcorr = repeated measures correlation; B2 = baseline day 2; SR1-SR5 = sleep restriction days 1 through 5; R1-R4 = recovery days 1 through 4; TSD = total sleep deprivation; ALL = all study days; CI = bootstrapped 95% confidence interval; Mdn = median; IQR = interquartile range.*

**TABLE 4:** PVT-10 and PVT-3 1/RT rmcorr results by time point.

Condition	Statistic	All study days					Recovery only				
Time		1000 h	1200 h	1600 h	1800 h	2000 h	1000 h	1200 h	1600 h	1800 h	2000 h
A	*r*_*rm*_	0.748	0.698	0.697	0.565	0.651	0.408	0.463	0.377	0.296	0.400
	*df*	387	409	405	408	383	100	122	119	121	122
	*p*	<0.001	<0.001	<0.001	<0.001	<0.001	<0.001	<0.001	<0.001	0.001	<0.001
	CI low	0.701	0.634	0.622	0.491	0.566	0.242	0.314	0.150	0.130	0.227
	CI high	0.791	0.759	0.760	0.636	0.724	0.578	0.606	0.570	0.439	0.564
PVT-10	Mdn (IQR)	3.61 (0.86)	3.64 (0.86)	3.65 (0.78)	3.51 (0.78)	3.54 (0.86)	3.69 (0.75)	3.73 (0.80)	3.64 (0.71)	3.55 (0.64)	3.58 (0.72)
PVT-3	Mdn (IQR)	3.89 (1.05)	3.88 (1.02)	3.93 (1.05)	3.93 (1.05)	3.91 (1.14)	4.03 (0.91)	4.01 (0.91)	4.03 (1.01)	3.94 (0.90)	4.03 (1.05)
B	*r*_*rm*_	0.824	0.784	0.792	0.736	0.808	0.506	0.354	0.449	0.632	0.449
	*df*	419	418	419	419	389	125	124	125	125	125
	*p*	<0.001	<0.001	<0.001	<0.001	<0.001	<0.001	<0.001	<0.001	<0.001	<0.001
	CI low	0.782	0.740	0.749	0.682	0.766	0.356	0.173	0.265	0.290	0.321
	CI high	0.860	0.822	0.827	0.788	0.845	0.652	0.519	0.589	0.772	0.586
PVT-10	Mdn (IQR)	3.71 (0.81)	3.72 (0.68)	3.71 (0.78)	3.67 (0.76)	3.69 (0.71)	3.90 (0.72)	3.89 (0.71)	3.86 (0.72)	3.82 (0.80)	3.84 (0.81)
PVT-3	Mdn (IQR)	3.91 (1.03)	3.94 (0.87)	3.95 (0.98)	3.97 (0.95)	3.96 (0.95)	4.15 (0.93)	4.17 (0.91)	4.20 (0.89)	4.17 (0.99)	4.12 (0.97)

*PVT-10 = 10-min Psychomotor Vigilance Test; PVT-3 = 3-min Psychomotor Vigilance Test; 1/RT = response speed; rmcorr = repeated measures correlation; CI = bootstrapped 95% confidence intervals; Mdn = median; IQR = interquartile range.*

#### By Study Phase

For PVT 1/RT rmcorr analyses across study phases, Condition B generally yielded stronger correlations relative to Condition A, but all by study phase analyses were significant for both conditions (Table 3). Only the all-study days *r*_*rm*_ was in the strong range for Condition A whereas the all-study days, B2-R4, and SR1-SR5 values were all in the strong range for Condition B. For Condition A, the B2-R4 and SR1-SR5 values were in the moderate range while the R1-R4 correlation was in the weak range. The R1-R4 correlation was also in the weak range for Condition B. Figure 4 presents examples of rmcorr plots for the SR1-SR5 and R1-R4 analyses for Condition A and Condition B.

#### By Day

The by day rmcorr analyses revealed a wide range of *r*_*rm*_ values for PVT 1/RT across study days, and all correlations were significant (Table 3). Condition B demonstrated strong magnitude correlations for SR1 and SR2 while a strong correlation was observed for Condition A on SR1. Moderate correlations were observed for TSD in both conditions, for SR2-SR4 and R1 for Condition A, and for SR3-SR5 and R4 for Condition B. R2-R4 and R1-R3 correlations were in the weak range for Conditions A and B, respectively. The SR5 correlation also was in the weak range for Condition A and the B2 correlation was in the weak range for Condition B. The only correlation in the negligible range was B2 for Condition A. Figure 5 presents B2, SR5, TSD, and R4 rmcorr plots for Condition A and Condition B.

#### By Time Point

The entire study (all-study) duration time point rmcorr analyses for PVT 1/RT were all significant for Condition A and Condition B (Table 4). Every *r*_*rm*_ coefficient for the all-study analyses was strong for Condition B while the 1000, 1200, and 1600 h time points had strong correlations for Condition A [the remaining time points, 1800 and 2000 h, had moderate correlations]. The recovery (R1-R4) time point *r*_*rm*_ coefficients were weaker than the all-study time points. For Condition A, all R1-R4 time point correlations were in the weak range. For Condition B, the R1-R4 1000 and 1800 h time point correlations were in the moderate range while the 1200, 1600, and 2000 h time point correlations were in the weak range. Figure 6 presents rmcorr plots for the 1800 h by time point analyses as an example of weak to strong magnitude rmcorr PVT 1/RT correlations by time point across the entire study and across recovery for Condition A and Condition B.

## Discussion

This is the first study examining the convergent validity of the PVT-3 relative to the “gold standard” PVT-10 across two commonly experienced sleep loss types followed by an extended recovery period when administered on the same device. Correlations for PVT 1/RT were stronger relative to PVT lapses throughout the study, yet both metrics were not strongly correlated consistently throughout SR and TSD. Notably, PVT-3 lapses and 1/RT both demonstrated poor correlations with the respective PVT-10 measures during baseline and recovery periods, when participants were not undergoing experimentally induced sleep loss. Generally, the PVT-3 demonstrated inadequate convergent validity (it failed to show *r*_*rm*_ values > 0.70, strong to very strong correlations indicative of adequate convergent validity) on two frequently used PVT metrics with the “gold standard” PVT-10 across baseline, SR, TSD, and extended recovery, and when considered by individual study day, by study phases, or by specific time points.

We hypothesized that rmcorr analyses would show relatively strong correlations between the PVT-10 and PVT-3 across all study phases of the sleep deprivation study on two frequently utilized PVT outcome metrics. Contrary to our expectations, only two *r*_*rm*_ coefficient values (out of 50; 4%) were above 0.70 for PVT lapses (both of those occurred in Condition B) while only 12 *r*_*rm*_ coefficient values (24%) were above 0.70 for PVT 1/RT (ten of those occurred in Condition B). Examined more granularly by time point, only analyses including all-study days for PVT 1/RT had *r*_*rm*_ values above 0.70, with Condition A only having one time point (1000 h) above this value, while no recovery time point analyses had *r*_*rm*_ values above 0.32 for PVT lapses or *r*_*rm*_ values above 0.63 for PVT 1/RT. Given that convergent validity coefficients less than 0.70 (less than strong or very strong) indicate validity issues (Carlson and Herdman, 2010; Post, 2016), these findings suggest that the convergent validity of the PVT-3 compared with the PVT-10 is inadequate based on two commonly used outcome metrics. Notably, our results are in line with, and expand upon, the findings of Jones et al. (2018) who concluded that the measures were not interchangeable.

Considered across the study, correlations for PVT 1/RT were generally stronger than those for PVT lapses, thus supporting our hypothesis. Our results correspond with previous studies that found PVT lapses more consistently differed and demonstrated lower correlations between the PVT-10 and PVT-3 relative to PVT 1/RT (Basner et al., 2011; Grant et al., 2017; Benderoth et al., 2021). Of note, although Grant et al. (2017) and Benderoth et al. (2021) found significant correlations between the PVT-3 and PVT-10 for 1/RT and lapses, the majority of correlations for lapses were below 0.70 and therefore similarly suggestive of validity issues. Interestingly, Basner et al. (2011) and Grant et al. (2017) attributed the observed differences between the measures to the use of different devices for administration [which notably is also similar to the Benderoth et al. (2021) study], yet the tests were administered on the same device in the present study [and in Jones et al. (2018)]; thus, the use of distinct devices does not likely explain any observed measure differences. The difference in lapse thresholds between the two PVT versions may also have potentially influenced the weaker correlations. While the thresholds we utilized (>500 ms for the PVT-10 and >355 ms for the PVT-3) are the most widely accepted thresholds in the sleep and circadian field, it could be valuable to investigate whether different lapse thresholds may better capture consistencies between the PVT-10 and the PVT-3 in future work.

Our hypothesis that correlations would be strongest during sleep deprived study phases compared to rested study phases (baseline and recovery) was generally supported. Correlations for recovery only time points, across recovery days, and across the recovery study phase were almost always weaker than those demonstrated during periods of sleep deprivation, while baseline correlations were non-significant, negligible, or weak. Since the Jones et al. (2018) study included multiple days of repeated measurements without sleep deprivation and determined the PVT-10 and PVT-3 measures did not perform comparably, our results provide further evidence that the lack of comparability is true for baseline measurements, and our results extend these findings to recovery periods following sleep loss interventions.

Different ISI durations between PVT versions could have potentially contributed to differential sensitivity to performance during rested and sleep deprived periods. One study failed to find a differential impact on PVT-10 and PVT-3 performance but reported that TSD enhanced the ISI effect (Yang et al., 2018). Since no study has evaluated the impact of differing ISIs on PVT performance in SR, it is unclear if SR would produce similar effects. If a differential impact of SR on the ISI effect were to be demonstrated, that factor, in tandem with incomplete recovery of PVT performance following SR (Yamazaki et al., 2021b) could explain the generally stronger correlations observed for those exposed to TSD first (Condition B) relative to those exposed to SR first (Condition A). Future research on the potential differential impact of SR relative to TSD on the ISI effect, the severity of incomplete recovery from SR, and the effects of sleep loss exposure order on neurobehavioral performance may provide insight into how these various factors interact.

The fact that the PVT-10 and PVT-3 are not comparable in their ability to measure behavioral attention during rested periods has implications for study design, test selection, and use of biomarkers or predictors relating to performance (Dawson et al., 2014; Basner et al., 2015; Grandner et al., 2018; Moreno-Villanueva et al., 2018). Moreover, studies that utilize baseline PVT-3 lapses or 1/RT to evaluate change with sleep loss should be interpreted with caution since reliance on such metrics for baseline comparisons is likely to yield misleading results. Given the importance of individual differences in neurobehavioral performance in sleep research (Leproult et al., 2003; Van Dongen et al., 2004; Tucker et al., 2007; Van Dongen, 2012; Spaeth et al., 2014; Ramakrishnan et al., 2015; Rusterholz et al., 2016; Dennis et al., 2017; Goel, 2017; Tkachenko and Dinges, 2018; Hajali et al., 2019; Letzen et al., 2019; Yamazaki and Goel, 2020; Brieva et al., 2021; Casale et al., 2022; Yamazaki et al., 2022a), it is essential that studies evaluating change over time, including in neurobehavioral response to sleep loss, are able to accurately assess baseline performance (Glymour et al., 2005). If PVT-3 and PVT-10 lapses and/or 1/RT outcome metrics are different when individuals are not impaired yet are slightly more comparable during periods of sleep deprivation, then it is possible (if not likely) that studies evaluating change over time that include baseline or recovery measurements would find disparate results depending on these measures.

Lastly, our hypothesis that correlation patterns across the extended recovery study phase would not differ between those exposed to TSD relative to those exposed to SR was not supported. Interestingly, correlations between the measures on R1 for those exposed to SR first (Condition A) were moderate while they were non-significant and negligible to weak for those exposed to TSD first (Condition B). Correlations on R2-R3 were comparable between conditions, yet R4 demonstrated negligible to weak correlations for Condition A, but these were moderate-to-strong for Condition B. Given that research on performance throughout extended recovery periods is limited, interpretation of these findings is challenging, but might relate to a differential recovery neurobehavioral performance profile following SR relative to TSD. Indeed, this is in line with a study from Yamazaki et al. (2021b) that found PVT-10 impairments resulting from SR did not fully recover even after extended recovery (four 12 h TIB nights), yet impairments fully recovered following TSD (Yamazaki et al., 2021a). Further studies are needed to explore these sleep loss-recovery dynamics.

Our study had a few limitations. These findings may not apply to different duration versions of the PVT such as the PVT-5 (Loh et al., 2004; Roach et al., 2006; Arsintescu et al., 2019) or to handheld versions of the PVT (Lamond et al., 2008). It is also possible that test administration order (i.e., in our study the PVT-10 always occurred before the PVT-3) may have impacted response of one or both measures, including potential time-on-task effects, though one study did not find evidence to support such an order effect (Basner et al., 2011). Future studies should examine the potential influence of order of test administration when assessing the convergent validity of PVT versions on the same device, as previous studies, including our own, did not examine this important possibility. In addition, our study did not assess system latency bias (the delay in response time due to test administration hardware and software platforms), which might have impacted our results, particularly for PVT lapses (Basner et al., 2021). Because the PVT-10 and PVT-3 were administered during the same testing session and on the same device during each test bout, any impact such bias had on PVT 1/RT should have been comparable between measures.

The highly controlled nature of our study, the large sample, the same device administration and the ability to utilize all available study data are unique strengths in the context of an evaluation of convergent validity. In our study, although PVT 1/RT specifically demonstrated relatively strong correlations across SR and TSD, most correlations were below an acceptable threshold for the measures to be considered interchangeable, while such correlations for PVT lapses were consistently below that threshold. Our results, coupled with the discordant results between the PVT-10 and PVT-3 observed during sleep loss on at least one major outcome metric in prior PVT-3 studies (Basner et al., 2011; Grant et al., 2017; Jones et al., 2018), indicate that the PVT-3 and PVT-10 may be measuring explicitly different constructs even during periods of impaired functioning. Thus, based on our experimental protocol and findings, the PVT-3 should be interpreted with caution when compared with the PVT-10 for lapses and 1/RT metrics. Since the PVT-3 has been proposed as a test to capture sleep loss-induced deficits rapidly and reliably in applied settings, such as aviation and other transportation sectors (Dawson et al., 2014), hospital shift work (Behrens et al., 2019), and security-related situations (Basner and Rubinstein, 2011), it is critical to discern whether the shorter 3-min version is a valid assessment of vigilant attention under both sleep-deprived and rested conditions. If so, this would allow for a rapid evaluation of objective vigilant attention in various operational settings. Other shorter alternatives to the PVT-10 such as the 5-min PVT version (Loh et al., 2004; Roach et al., 2006; Arsintescu et al., 2019) are available for use in applied settings, yet Jones et al. (2018) found differences between 5- and 10-min PVT; as such, future studies including those involving sleep loss in applied settings are needed. This work could evince the utility of time-efficient objective metrics in assessing an individual’s level of vigilant attentional impairment from sleep loss.

## Data Availability Statement

The data generated and analyzed during the current study are available from the corresponding author upon reasonable request.

## Ethics Statement

The studies involving human participants were reviewed and approved by the University of Pennsylvania’s Institutional Review Board. The participants provided their written informed consent to participate in this study.

## Author Contributions

NG designed the original study during which the analyzed data were collected and extracted and retains oversight and control of all data as Principal Investigator. NG provided financial support, including all research grant support, all personnel support, and all publication fees. CA conceived of the present study, identified and verified the statistical methods, and performed the analyses. EY, CC, and TB checked analysis scripts, plots, and results for accuracy. CA wrote the manuscript. All authors contributed to the interpretation of the results, provided critical feedback, helped shape the research, analysis, and manuscript, and reviewed and approved the final manuscript.

## Conflict of Interest

The authors declare that the research was conducted in the absence of any commercial or financial relationships that could be construed as a potential conflict of interest.

## Publisher’s Note

All claims expressed in this article are solely those of the authors and do not necessarily represent those of their affiliated organizations, or those of the publisher, the editors and the reviewers. Any product that may be evaluated in this article, or claim that may be made by its manufacturer, is not guaranteed or endorsed by the publisher.
